# Epidemiology of burn injuries in the East Mediterranean Region: a systematic review

**DOI:** 10.1186/1471-2458-10-83

**Published:** 2010-02-20

**Authors:** Nasih Othman, Denise Kendrick

**Affiliations:** 1 School of Community Health Sciences, University of Nottingham, Nottingham, UK

## Abstract

**Background:**

Burn injuries remain one of the leading causes of injury morbidity and mortality in the World Health Organization's East Mediterranean Region. To provide an overview on the epidemiology of burn injuries in this region, a systematic review was undertaken.

**Methods:**

Medline, Embase and CINAHL were searched for publications on burns in this region published between 01/01/1997 and 16/4/2007. Data were extracted to a standard spreadsheet and synthesised using a narrative synthesis. No attempt has been made to quantitatively synthesise the data due to the large degree of clinical heterogeneity between study populations.

**Results:**

Seventy one studies were included in the review, from 12 countries. Burn injuries were found to be one of the leading causes of injury morbidity and mortality. The reported incidence of burns ranged from 112 to 518 per 100,000 per year. Burn victims were more frequently young and approximately one third of the victims were children aged 0-5 years. Hospital mortality ranged from 5 to 37%, but was commonly above 20%. Intentional self-harm burns particularly involving women were common in some countries of the region and were associated with a very high mortality of up to 79%.

**Conclusion:**

Burn injuries remain an important public health issue in the East Mediterranean Region therefore further research is required to investigate the problem and assess the effectiveness of intervention programmes.

## Background

The World Health Organization's (WHO) East Mediterranean Region (EMR) covers a population of over 500 million, spread over a wide area of relative cultural and geographical similarity extending from Morocco to Afghanistan. The EMR includes 22 countries; Afghanistan, Bahrain, Djibouti, Egypt, Iran, Iraq, Jordan, Kuwait, Lebanon, Libya, Morocco, Oman, Pakistan, Palestine, Qatar, Saudi Arabia, Somalia, Sudan, Syria, Tunisia, United Arab Emirates and Yemen. Of these countries Kuwait, Qatar and United Arab Emirates are classified as high-income countries and the rest are classified as low-income or middle-income countries[1].

According to the WHO's International Classification of Diseases version 10(ICD-10), burn injuries are classified by site of injury in chapter XIX as "burns and corrosions" (T20-T32) and in terms of aetiology, they are classified as those caused by exposure to smoke, fire and flames (X00-X09), contact with heat and hot substances (X10-X19), exposure to electric current (W85-87), lightening(X33) and exposure to corrosive substances (X46, X49). Therefore burns include scalds as wells as injuries caused by heat from electrical heating appliances, electricity, flame, friction, hot air and hot gases, hot objects, lightening and chemical burns (both external and internal corrosions from caustic chemicals). Radiation-related disorders of the skin and subcutaneous tissue and sunburn are not included in this classification of burns [2].

Burn injuries are a major problem in the low-income and middle-income countries. The WHO estimates indicate that globally there were more than 7.1 million fire-related unintentional burns (X01-X09) in 2004 giving an overall incidence rate of 110 per 100,000 per year. The incidence in the EMR was 187 per 100,000 per year compared to the lowest incidence in the Americas which was 19 and the highest incidence in South East Asia which was 243 per 100,000 per year[3]. The WHO estimates that 310,000 people died in fires in 2004 across the world, the great majority being in low-income and middle-income countries with a global mortality rate amounting to 4.8 per 100,000 per year[4]. According to these WHO data, 29,000 deaths occurred in the EMR with a mortality rate of 5.6 deaths per 100,000 [4].

Published literature indicates that burn injuries remain a major health problem in the EMR countries, although there has been no published attempt to summarise this literature to date. In view of the cultural, economic and geographic similarities among the countries of this region, summarising the literature will describe the extent of the problem, and highlight common features and differences regarding the epidemiology of burn injuries. Such information will be useful for planning prevention strategies and identifying further research questions that need to be answered.

## Methods

This systematic review was undertaken to describe the epidemiology of burns in the EMR between the years 1997 and 2007. All published studies relevant to the epidemiology of burns in the region were considered for inclusion in the review. The main outcomes included the incidence of burns, mechanism of burns and mortality.

### Inclusion/exclusion criteria

Studies investigating the epidemiology of burns in the countries of the region were included if they were published between 1997 and 2007 using methodologies including cross sectional surveys, retrospective and prospective studies, systematic reviews and case-control studies. The following types of articles were excluded: 1) articles about specific aspects of burn management; 2) methodologies apart from those mentioned above e.g. case reports, editorials etc.; 3) military and war related burns and 4) articles repeating data from other articles already included.

### Search strategy

Medline, Embase and CINAHL were searched for publication dates between 01/01/1997 and 16/4/2007. The search strategy included the following terms: burn*, scald*, thermal injur*, combined by OR; AND the names of all 22 countries of the region combined by OR. In addition a manual search was undertaken of the WHO's East Mediterranean Health Journal from its website. Articles in all languages were retrieved.

### Selection of the studies

**Figure 1:**
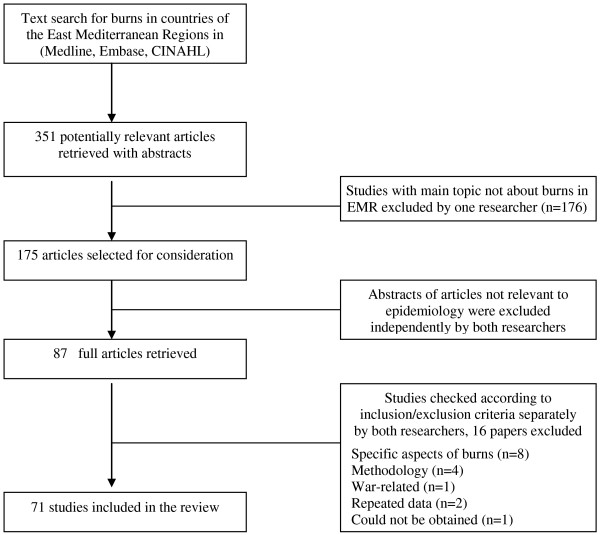
**Selection of studies for inclusion in the review**.

The search strategy retrieved 351 potentially relevant articles with abstracts (see figure 1). One researcher (NO) reviewed the abstracts and excluded studies which were not about EMR countries or the main topic was not about burns. The titles and or abstracts of the remaining 175 articles were assessed for inclusion independently by both researchers to select those relevant to epidemiology of burns. Eighty seven articles were selected and their full texts were obtained including some in French and Persian. Where these had English abstracts they were assessed for inclusion by 2 reviewers, otherwise they were assessed for inclusion and data were extracted by one reviewer who was fluent in these languages. Using the inclusion/exclusion criteria, the two researchers independently reviewed these articles and finally selected 71 studies for inclusion in the review. Any disagreement between the two reviewers about inclusion was settled by discussion.

### Data extraction

Data were extracted from full copies of published articles using a standard data extraction spreadsheet. Data were extracted from the first 20 articles by both reviewers independently and compared to identify discrepancies. Consistency of data extraction between the 2 researchers was assessed by comparing the accuracy of the information retrieved by both of them against the original data in the study. In 98% of cases they had retrieved identical and accurate information. Then one researcher (NO) extracted data from the remaining studies.

### Quality of studies

The quality of included studies was assessed using a modification of checklists described by Downs et al[5] and Macfarlane et al[6]. This checklist was developed further to make it feasible to apply to studies with the range of methodologies included in this review. The checklist included 20 items to assess different stages of the research and report writing. The reviewers discussed and agreed how to apply each item to studies, and then 20 studies were assessed by both reviewers and agreement was measured. The agreement between the two reviewers on individual items ranged from 70% to 100% and kappa coefficients ranged from 0.35 (P = 0.037) to 1.0 (P < 0.001). Following this, further discussions were held between the reviewers on the application of the checklist. Quality for the remaining studies was assessed by one reviewer (NO)

Data have been synthesised using a narrative synthesis. No attempt has been made to quantitatively synthesise the data due to the large degree of clinical heterogeneity between study populations.

## Results

Of the 351 articles initially retrieved, 71 studies were included in the review which came from 12 of the 22 countries of the region. No studies were found from Bahrain, Djibouti, Lebanon, Libya, Palestine, Qatar, Somalia, Sudan, Syria and Yemen. Fifty five of the studies specifically described the epidemiology of burns, whilst 16 described the epidemiology of a range of injuries, including burns. As shown in table 1, most studies came from Iran (44%), Saudi Arabia (13%) and Egypt (10%). Thirty studies were published in the first five years of the period under review (1997-2001) and 39 studies were published in the second half of the period (2002-2006). Most studies (62%) used survey or retrospective methodology and 38% used prospective methodology. There were no case control studies.

**Table 1:** Main characteristics of studies included in the review (n = 71)

Characteristic	Number of studies	Percent
Country		
Iran	31	44
Saudi Arabia	9	13
Egypt	7	10
Kuwait	6	9
Pakistan	6	9
Tunisia	3	4
Jordan	2	3
Iraq	2	3
UAE	2	3
Afghanistan	1	1
Morocco	1	1
Oman	1	1
Year of Publication		
2007(first 3 months)	2	3
2006	11	15
2005	8	11
2004	8	11
2003	4	6
2002	8	11
2001	3	4
2000	6	8
1999	3	4
1998	7	10
1997	11	15
Study design		
Survey/retrospective	44	62
Prospective	27	38
Setting		
Hospital	62	87
Community	8	11
Forensic records	1	1
Patient type		
Only admissions	46	65
Admissions & outpatients	15	21
Only outpatients	10	14
Injury type		
Burns only	55	77
All injuries	16	23

### Quality of studies

The quality of studies was assessed by the checklist shown in table 2. Most studies reported objectives and outcomes, described the research setting and presented the results clearly. Few studies elaborated on sample size calculation and justification (14%), representativeness (14%), response rate (13%), limitations of retrospective records (12%), description of non-participants (9%) and limitations of the study in the discussion section (23%).

**Table 2:** Assessment of the quality of included studies

Item	Number of studies with criteria (%)
1. Hypothesis/aims of the study clearly stated	71 (100)
2. Main outcomes clearly described in the introduction/methods	71 (100)
3. Study design clearly described	62 (87)
4. Setting of the study clearly described	63 (88)
5. Source of the subjects clearly described	36 (51)
6. Sample size calculation stated and justified	7 (14)
7. Sample representative of the target population	10 (14)
8. Participation/response rate stated	9 (13)
9. (Retrospective studies) Study covers all the records of the specified time	31 (72)
10. (Retrospective studies) Limitations of the records described	5 (12)
11. (Prospective studies) Strategies described to improve participation/follow up	2 (7)
12. Non-participants/subjects lost to follow up described	4 (9)
13. Exposures accurately measured to minimize bias	67 (94)
14. Outcomes accurately measured to minimize bias	69 (97)
15. Results clearly described	71 (100)
16. Statistical methods sound and justified	68 (96)
17. P-values reported	42 (59)
18. Confidence intervals reported	44 (62)
19. Limitations of the study described	17 (23)
20. Main outcome measurements can be considered valid	71 (100)

### Burns as a major cause of injury morbidity and mortality

Surveillance of injuries in Iran has found that burns are the most common cause of unintentional home-related injuries accounting for 40% of those injuries in all ages[7]. Another survey from Iran reports that 12% of all deaths in all ages are from unintentional injuries and burns are the second most common cause of injury-related deaths after road traffic accidents[8]. This finding is supported by a review of forensic records from Tehran reporting that burn injuries account for 18% of unintentional deaths in children aged 15 years or less, second only to road traffic accidents[9]. A survey in rural areas of Iran also reports similar findings; 12% of all childhood unintentional injury deaths and 10% of all-age unintentional injury deaths were due to burn injuries[10,11]. In the United Arab Emirates, burns are responsible for 9% of all childhood injuries and 14% of childhood injury deaths[12] being the third most common cause of injury mortality [13]. Similar findings are reported by studies elsewhere in the region [14,15].

### Incidence

The annual incidence of burns, the incidence of hospital admissions and the incidence by gender have been reported by several studies. All age incidence of burns ranges from 112 to 518 per 100,000 per year [16-22]. A much higher incidence of 1,388 per 100,000 per year is reported amongst children below 5 years by a study from Pakistan[22]. A higher incidence of burns is also reported in squatter settlements of Karachi[23]. The annual incidence of burn admissions in all ages is reported by some studies with higher rates in females including 13.4 per 100,000 per year[19], 13.5 (male 9.1, female 18.0)[16], 17.2[18] and 19,0 admissions per 100,000 per year (male 15.5, female 18.9)[24].

Reported admission rates amongst children aged 0-15 years range from 11.8 to 20.8 per 100,000 per year[25-27] with higher rates in younger children and lower rates in older children. For example, reported rates include 36.9 per 100,000 per year in children aged 2 years in Iran[27], 34.0 per 100,000 per year in children aged 0-4 years in Kuwait[26], 22.3 in children aged 0-5 years in Iran[18] and 5.9 per 100,000 in children aged 6-10 years in Iran[18]. According to these studies admission rates are higher in boys of younger ages such as 25.5 per 100,000 per year amongst boys aged 0-5 years and 18.8 in girls of the same age[18]; 45.8 in boys aged 2-3 years and 28.1 in girls of the same age[25]. Amongst older children, admission rates are higher in girls, according to a study from Iran reporting admission rates of 7.3 per 100,000 per year in boys aged 11-15 years versus 13.2 in girls of the same age[18].

### Age and sex in burn injuries

Additional file 1 shows the age and gender distribution in studies reporting these characteristics. The overall mean age varies depending on the age range of participants and type of burns included, but when all ages are included most studies have reported means ages between 18 and 25 years. The majority of burns in childhood occur in the 0-5 year age group, which comprises up to 78% [28] of all childhood burns and up to 38% of burns in all ages [17]. In terms of gender in children, all studies report a higher proportion of males suffering burns compared to females. Regarding all ages, many Iranian studies report a higher proportion of females suffering burns compared to males while others studies report a higher proportion of males (Additional file 1).

### Mechanism of burn injuries

Additional file 1 summarizes the mechanisms of burns according to different studies. Overall, flame injuries are generally more common than scalds amongst admitted patients. However, a community-based study of all accidental burns (both medically attended and not attended) found that 75% of burns were scalds, 16% were due to flames, 10% were due to contact with hot objects and 2% were electrical[17]. Many studies report a higher proportion of flame injuries than scalds amongst admitted patients [18-21,29-34]. Fewer studies report a similar or a higher proportion of scalds than flame injuries [35-37]. Amongst children, all studies report that scalds are more common than flame injuries (Additional file 1).

Contact burns are reported by a few studies with the highest proportion being 13% of all burns[37]. In terms of chemical burns, external corrosions (contact with the skin) comprise 1% to 4% of all burns [17,21,25,28,32,38,39]. According to a study exclusively on these chemical burns , 75% of cases were due to sodium hydroxide drain cleaners, 11% due to acid substances and 4% due to application of herbs used as traditional medication[40]. Ingestion of caustic material is another cause of chemical burns in children 0-14 years as reported by 4 studies[41-44]. In these studies males comprise 57-60% of the sample. The causative agents for these chemical burns are largely ingestion of alkali compounds accounting for 85% [41] and 89%[43] of the burns. Acids account for 9% and 7% respectively. Electrical burns comprise 27% of all burns in an Egyptian study [34] involving a high proportion of work-related injuries but generally their contribution is much less (Additional file 1).

### Place of burn injuries

All studies that have reported on the place of the incident indicate that burns most commonly occur at home ranging from 72% [25] to 94% [19]. One study reported that 99% for childhood scalds occurred at home[45]. Older children were significantly more likely to be burnt outside home compared to younger children [25,27].

### Season of burn injuries

In almost all studies winter is the commonest season for burn occurrence. Winter accounts for 28-31% of burns in several studies [18,19,24,34,38,46]. Some studies have reported even higher proportions (44%) of burns occurring during winter in children [39] and older people [47].

### Burn size

The percent total body surface area (TBSA) burnt in admitted patients ranges from 1-100%. The mean TBSA burnt in all ages ranges from 10 to 48% and in children from 14-30%. The TBSA burnt for females is consistently higher than that for males (Table 3).

**Table 3:** Mortality, TBSA burnt and hospital stay in included studies

Year	Country	Patients	Mortality %	Mean (Median) %TBSA burnt			Stay in days Mean (median)
					
				All	Deaths	Survivors	
1998	Iran[48]	All ages	37.0	38	-	-	12.0
2001	Iran[19]	All ages	34.4	42 (35)	67 (67)	27 (25)	-
2002	Iran[18]	All ages	33.0	48 (40)	73 (88)	-	13.0 (9.0)
1997	Egypt[29]	All ages	33.0	-	-	-	16.2 (19.5)
2006	Pakistan[35]	All ages	30.0	-	-	-	-
2005	Iran[24]	All ages	21.3	-	-	-	-
2005	Iran[20]	All ages	18.7	26	65	17	12.0
2002	Afghanistan[36]	All ages	16.0	19 (15)	-	-	11.0 (7.0)
1997	Kuwait[69]	All ages	6.4	-	70	20	16.0
1997	Saudi Arabia[33]	All ages	5.6	23	-	-	-
2005	Kuwait[32]	All ages	5.3	10	80	10	-
2002	Iran[67]	>6 years	37.0	38	-	-	16.0
2002	Iran[27]	Children	17.2	30	-	-	-
2001	Iran[25]	Children	16.0	26 (23)	48	22	15.8
2005	Iran[50]	Children	6.4	19	-	-	-
1997	Saudi Arabia[71]	children	2.8	15	70	-	20.4
2006	Kuwait[26]	Children	1.3	(13)	-	-	14.6
2004	Saudi Arabia	Children	1.0	-	-	-	8.5
1997	Kuwait	Children	1.0	14	-	-	16.9
2003	Egypt[47]	Elderly	49.0	22	-	-	22.0
2006	Iran[57]	Pregnant	39.2	38	69	18	-
1997	Egypt[72]	Women	39.0	-	-	-	-

### Mortality and hospital stay

The mortality in relation to burn size and hospital stay is shown in Table 3. A Kuwaiti study [32] reported an all age mortality rate of 0.6 per 100,000 per year while two Iranian studies have reported a much higher mortality rate of 4.6 [19] and 5.6 [20] per 100,000 per year. In children 0-15 years the reported rates include 0.2 [32], 2.0 [27] and 3.2 deaths per 100,000 per year[25].

The in-hospital mortality for all burn injuries amongst all ages ranges from as low as 5% (mean TBSA burnt = 10%) in Kuwait[32] to 37% (mean TBSA burnt = 38%) in Iran[48]. The in-hospital mortality exceeds 20% in many studies. Mortality in children ranges from 1% (mean TBSA burnt = 14%) in Kuwait [45] to 17% (mean TBSA burnt = 30.2%) in Iran [27].

Factors associated with mortality according to individual studies include older age (60 and over), a greater TBSA burnt, female gender, depth of burn and delay in receiving medical care[29]; TBSA burnt and age [33]; inhalation, delay and female gender [49], TBSA burnt and inhalation[50], TBSA burnt, flame, female gender and age[18]; and TBSA burnt and head and neck burns [51]. Mortality for flame injuries is much higher than for scald injuries. While flame burn mortality rates are reported as 42% [35] and 44%[24] in all ages and 31% in children[27], mortality rates for scald injuries are reported by the same authors as 11%, 5% and 4% respectively.

### Burn in particular populations

A study on accidental burns in adult epileptics[52] occurring during a convulsion, reports that 90% of burns happened at home, 70% were flame injuries and 30% were scald injuries. Another study reports that children 1-7 years with congenital brachial palsy are at risk of contact burns[53]. Twelve males including children are reported sustaining foot burns from prolonged contact with hot ground during Friday prayers in Saudi Arabia[54]. Another unusual study on friction burns from car tyre injuries reports on 23 children and young individuals (80% of them males) who sustained friction burns from car accidents [55]. A Kuwaiti study reports on burning as a method of physical abuse of children and says that 38% of such abuses were done through burning[56]. A study on burns during pregnancy [57] with mean TBSA burnt of 38%, reports maternal and foetal mortalities of 39% and 45% respectively.

### Intentional self-harm

Burn injuries appear to be a common method of deliberate self-harm in some countries of this region. In Iran, burns are responsible for 22% (male 14%, female 31%) of all suicide attempts and 17% (male 9%, female 26%) of suicide deaths[58]. Another study reports that burns are responsible for 41% of all suicide deaths in Iran [59]. Table 4 lists studies reporting on intentional self-harm burns. The incidence of intentional self-harm burns as reported from different provinces of Iran ranges from 2.9 to 21 per 100,000 per year. The TBSA burnt is higher than in accidental burns, the mean TBSA burnt ranging from 45 to 76%. The mortality is also expectedly high in correspondence with the TBSA burnt ranging from 56 to 80% (Table 4).

**Table 4:** Incidence, sex, age, TBSA burnt and mortality in intentional self-harm burns

Year	Country	Sample	Incidence	Sex %		Age		TBSA mean	Mortality %
			/100,000						
				Male	Female	Range	Mean (median)		
1997	Egypt[73]	23	-	9	91	14-55	23.0	45	74.0
2002	Iran[74]	318	8.2	17	83	-	27.0	63	79.0
2003	Iran[75]	110	-	100	-	14-68	26.9(25)	76	77.0
2004	Iran[61]	412	12.5	1	99	15-72	25.5	66	79.6
2005	Iran[62]	35*	-	-	100	15-35	24.0	-	-
2005	Iran[76]	98	7.7	24	77	11-68	27.0	63	76.0
2006	Iran[60]	358	6.5	26	74	-	-	-	65.5
2006	Iran[16]	54	18.1**	18	82	13-19	16.8	70	58.0
2006	Iran[51]	117	4.9	22	78	-	28.0	63	77.8
2007	Iran[77]	89	2.9	21	79	13-62	26.0(24)	63	56.0
2007	Iran[59]	37	2.1*	19	81	14-50	24.9		-

* Only deaths included in this study but rate is for self-harm burns

** Only Adolescents aged 13-19 years

Intentional self-harm is responsible for a variable proportion of burn admissions ranging from 2% in Pakistan[21] to as high as 37% [24] of all burn admissions in Iran. However, most proportions fall between 10 and 20% of all burn admissions.

The studies indicate that the victims of these burns are mostly young; with a mean age ranging from 17 to 27 years although they include individuals as young as 11 years and as old as 72. These victims are more frequently women comprising from 74%[60] to as high as 99% [61] of all intentional self-harm burn admissions. Intentional self-harm burns are flame injuries caused by kerosene in up to 91% of cases[62] and mostly occurring at home. The most frequently reported motives for these burns are marital problems. Other motives include psychological and psychiatric disorders, family problems, poverty and emotional relationships.

## Discussion

### Main findings

This systematic review summarizes the epidemiological characteristics of burn injuries in the EMR as reported by 71 different studies from 12 countries of the region during the period of 1997 to 2007. Burn injuries are one of the leading causes of injury morbidity and mortality. The reported incidence of burn injuries ranges from 112 to 518 per 100,000 per year. Burns most commonly occur in the young with mean ages below 25 years reported by most studies. Many studies report that more than 20% of admitted patients die in hospital. Mortality is lower in children but it is considerably more, up to 79%, in burns caused by intentional self-harm.

### Strengths and limitations of the study

We used a standard systematic review methodology including selection of studies for inclusion, data extraction and quality assessment of a sample of the studies by two independent reviewers. We restricted our review to the published articles indexed in Medline, Embase and CINHAL. We will therefore have not found unpublished studies or those published in journals not indexed in these databases. As with any systematic review, the data that can be extracted from studies is in part dependant on the quality of the reporting and in part on the quality of the research. Many studies failed to describe non-participants, report sample size calculations or response rates, or to discuss the representativeness of the study population, the use of retrospective records or limitations of the study. In addition, there was considerable heterogeneity in the way in which studies reported their findings which limited some of the comparisons we were able to make.

### Explanation of findings

Although there are differences in reported figures, it is evident that burn injuries remain a major cause of injury morbidity and mortality in the region especially amongst children and more so in home injuries [7-15,46]. Some of the variation in incidence rates, gender distribution, injury mechanism, TBSA burnt, mortality and other characteristics is likely to be related to differences in study designs, setting, study population and representativeness of individual studies.

There are other potential explanations for the observed variation. Firstly the study population in studies undertaken in specialized burns centres may not be representative of the population of people with burn injuries due to the severity of the burn, access to medical care, admission policies and traditional beliefs regarding home treatment of burns. Secondly, studies using retrospective data from hospital records are prone to the usual limitations of accuracy and completeness, hence incidence rates based on hospital data are likely to be underestimates of the true rate of medically-attended burn injuries. This is supported by the finding of higher estimates for the burden and incidence of burn injuries from community surveys than hospital-based studies[7,10,11,17,22,23,46].

It is clear that women and children, especially younger children, are at greater risk of burns and that most of these injuries occur in the home. This may be partly explained by women spending more time at home, participating in activities such as preparing and serving hot drinks, cooking and heating water and being exposed to equipment and devices such as space heaters and stoves. In addition to these exposures, the design and layout of the house, availability of safe places to play, developmental stage and supervision practices are likely to impact on burn risk for children.

Winter is the cold season in the region and in countries which do not have a regular supply of electricity or where electricity is less affordable than other means, households are likely to use kerosene devices for space heating and also for heating water and making hot drinks. Household members are also likely to spend a greater proportion of their time indoors in cold weather, which may help explain the winter excess of burn injuries, particularly amongst children[39] and older people [47].

The reported morality from burn injuries varies across studies, ranging from 5% to 37%. The data presented in the included studies does not allow conclusions to be drawn about the reasons why mortality rates show such a large degree of variation, other than variations in study populations, injury mechanisms and injury severity. Specifically conclusions cannot be drawn about the relationship between the quality of medical care and the mortality rate. It is clear from Table 3 that mortality is generally higher when the mean or median TBSA burnt is higher.

### Recommendations for research and for practice

Standardisation of the reporting of epidemiological studies of burn injuries would be helpful in improving the quality, at least of the reporting, of such studies. Reporting studies in line with the STROBE (Strengthening the Reporting of Observational studies in Epidemiology) guidelines[63] would address many of the deficiencies we found in the quality of the reporting of studies, and journals publishing epidemiological studies of burn injuries should require their use. In addition to using these guidelines, there is some information specific to burns that would be helpful to include in epidemiological studies of burn injuries. This includes reporting age groups in line with the WHO injury reports[1], reporting the distribution of the total body surface area burnt more extensively that merely providing the mean or median (e.g. at least report quartiles or quintiles) and reporting mortality by these categories of TBSA burnt.

The extent of the problem of burn injuries in the Eastern Mediterranean Region, in particular those occurring at home, those occurring in children and intentional burns in women, suggests that greater efforts are required to prevent such injuries. Further research is required to summarise the literature on the effectiveness of interventions to prevent thermal injuries that are common in this region and to make an assessment of the extent to which the evidence is translatable to the countries within the EMR. Following this, burn prevention strategies need to be devised and prevention programmes need to be developed implemented and evaluated that address specific burn injury mechanisms in these high risk settings and in these high risk groups.

## Conclusion

Burn injuries are an important public health issue in the East Mediterranean Region being one of the leading causes of injury morbidity and mortality. In addition, self-burning appears to be a common method of suicide amongst women in some countries of the region. Further research (which may have to allow for strategies to include locally published literature) is required to summarise the situation and the effectiveness of any preventive interventions undertaken, in order to provide evidence for developing and implementation of burn prevention programmes especially addressing high risk groups such as children and young females.

## Competing interests

The authors declare that they have no competing interests.

## Authors' contributions

NO contributed to the following stages of the study: conception of the idea of the research, undertaking literature search, data extraction, drafting the article, undertaking subsequent revisions and interpretation of the findings. DK contributed to the following stages of the study: overall supervision of the process, data extraction, critical review, interpretation of the findings and the final revision. Both authors have approved the final manuscript.

## Pre-publication history

The pre-publication history for this paper can be accessed here:

http://www.biomedcentral.com/1471-2458/10/83/prepub

## Supplementary Material

Additional file 1 **Table S1 - Age, sex and mechanism of burn injuries in studies reporting these characteristics**. The file contains Additional file 1 representing data on age, sex and mechanism of burn injuries. This is part of the results section. Click here for file
