# Supplementary material for: Epidemiology of burn injuries in the East Mediterranean Region: a systematic review
Source: BMC Public Health. 2010 Feb 20;10:83. doi: 10.1186/1471-2458-10-83 (PMC2841676; doi:10.1186/1471-2458-10-83)
Supplement: Additional file 1 — Table S1 - Age, sex and mechanism of burn injuries in studies reporting these characteristics. The file contains Additional file 1 representing data on age, sex and mechanism of burn injuries. This is part of the results section. [file 1471-2458-10-83-S1.DOC]

|  |  |  |  |  |  | **Sex %** | |  | **Age in years** | |  | **Mechanism of burn %** | | | | |
| --- | --- | --- | --- | --- | --- | --- | --- | --- | --- | --- | --- | --- | --- | --- | --- | --- |
| **Year** | **Country** | **Design*** | **Patients**** | **Burns** | **n** | **Male** | **Female** |  | **Range** | **Mean(median)** |  | **Flame** | **Scald** | **Contact** | **Chemical** | **Electrical** |
| *Study population: Children* | | | |  |  |  |  |  |  |  |  |  |  |  |  |  |
| 1998 | Egypt[39] | Pro | Both | All | 305 | 54 | 46 |  | 0-14 |  |  | 39.0 | 57.0 |  | 2.0 | 3.0 |
| 2005 | Iran[50] | Retro | In | All | 1160 | 61 | 39 |  | 0-14 | 2.2 |  | 30.5 | 66.6 |  |  | 1.3 |
| 2001 | Iran[25] | Retro | In | All | 1454 | 73 | 27 |  | 0-15 | 5.3 (4) |  | 35.7 | 56.0 |  | 0.5 | 3.9 |
| 2002 | Iran[27] | Retro | In | All | 760 | 58 | 42 |  | 0-15 | 7.1 (6) |  | 43.0 | 46.0 | 1.3 | 0.1 | 1.8 |
| 1998 | Jordan[41] | Retro | In | Chemical | 216 | 60 | 40 |  | 0-14 |  |  |  |  |  |  |  |
| 1997 | Kuwait[45] | Pro | In | Scalds | 388 | 60 | 41 |  | 0-12 | 3.0 |  |  |  |  |  |  |
| 2006 | Kuwait[26] | Pro | In | All | 826 | 64 | 36 |  | 0-14 | 4.1 (3) |  | 23.0 | 67.0 |  |  | 8.0 |
| 1997 | Morocco[64] | Retro | In | All | 59 | 58 | 42 |  | 0-12 | 3.5 |  | 41.0 | 54.0 |  |  | 3.0 |
| 2004 | Saudi Arabia[28] | Retro | In | All | 380 | 50 | 50 |  | 0-12 |  |  | 28.0 | 64.0 |  | 1.8 | 5.0 |
| 2004 | Tunis[65] | Pro | In | Chemical | 56 | 57 | 43 |  | 1-11 | 4.0 |  |  |  |  |  |  |
| 2004 | Tunis[42] | Retro | In | Chemical | 330 | 59 | 41 |  | 0-14 | 3.4 |  |  |  |  |  |  |
| *Study population: All ages* | | | |  |  |  |  |  |  |  |  |  |  |  |  |  |
| 2002 | Afghanistan[36] | Retro | In | All | 388 | 57 | 43 |  | 0-70 | 13 (8) |  | 37.0 | 44.0 |  |  | 2.0 |
| 1997 | Egypt[29] | Retro | In | All | 533 | 50 | 50 |  | 0-81 | 22.9 |  | 66.8 | 26.3 |  | 3.0 | 3.9 |
| 2000 | Egypt[66] | Pro | Both | Stove | 304 | 51 | 49 |  | 1-75 | 23.6 |  |  |  |  |  |  |
| 2003 | Egypt[34] | Pro | In | All | 880 | 47 | 53 |  | 0-75 | 27.8 |  | 41.0 | 32.0 |  |  | 27.0 |
| 1998 | Iran[48] | Retro | In | All | 1239 | 63 | 37 |  | 0-93 | 25.6 |  |  |  |  |  |  |
| 2001 | Iran[19] | Retro | In | All | 2043 | 44 | 56 |  | 0-98 | 21.9 |  | 76.0 | 17.0 |  | 2.0 | 3.0 |
| 2002 | Iran[67] | Retro | In | All | 1082 | 40 | 60 |  | 6-100 | 27.0 |  |  |  |  |  |  |
| 2002 | Iran[18] | Retro | In | All | 1089 | 34 | 66 |  | 0-90 | 20.6 |  | 64.0 | 24.0 |  |  |  |
| 2003 | Iran[37] | Retro | In | All | 1493 | 53 | 47 |  | 0-95 | 21.8 |  | 25.0 | 53.0 | 13.0 |  | 2.0 |
| 2005 | Iran[20] | Retro | In | All | 2963 | 56 | 44 |  | 0-89 | 22.0 |  | 55.0 | 36.7 |  |  | 3.0 |
| 2005 | Iran[24] | Retro | In | All | 235 | 41 | 59 |  | 0-85 | 19.4 |  |  |  |  |  |  |
| 2006 | Iran[17] | Survey | Out | All | 1179 | 41 | 59 |  |  | 18.8 |  | 16.2 | 74.9 | 9.8 | 0.7 | 1.5 |
| 2005 | Iraq[68] | Retro | In | All | 48 | 48 | 52 |  | 0-45 |  |  |  |  |  |  |  |
| 1997 | Iraq[30] | Pro | In | All | 127 | 46 | 54 |  | 1-67 | 20.0 |  | 63.0 | 22.0 |  |  | 11.0 |
| 1997 | Kuwait [31] | Retro | In | All | 1213 | 67 | 33 |  | 0-93 | 23.0 |  | 53.7 | 37.6 |  |  | 6.8 |
| 1997 | Kuwait[69] | Retro | In | All*** | 234 | 48 | 52 |  | 1-93 | 30.0 |  | 92.3 | 7.7 |  |  |  |
| 2005 | Kuwait[32] | Pro | In | All | 2111 | 70 | 30 |  | 1-94 | (25) |  | 52.0 | 37.8 |  | 1.3 | 7.8 |
| 1998 | Oman[70] | Retro | In | All | 168 | 58 | 42 |  |  |  |  |  |  |  |  |  |
| 1998 | Pakistan[21] | Retro | In | All | 716 | 49 | 52 |  | 1-85 | 23.0 |  | 67.4 | 20.7 | 4.2 | 1.9 | 2.6 |
| 2006 | Pakistan[35] | Retro | In | All | 111 | 55 | 45 |  | 0- | 19.0 |  | 41.0 | 40.5 |  | 10.0 | 5.0 |
| 1997 | Saudi Arabia[71] | Retro | In | All | 277 | 60 | 40 |  | 0-85 |  |  | 37.5 | 49.0 |  | 3.6 | 7.6 |
| 1997 | Saudi Arabia[33] | Retro | In | All | 90 | 51 | 49 |  | 0-55 | 15.0 |  | 52.0 | 40.0 |  | 4.4 | 3.3 |
| 2001 | Saudi Arabia[40] | Retro | In | Chemical | 59 | 75 | 25 |  | 2-70 | 25.0 |  |  |  |  |  |  |
| *Study population: Older people* | | | |  |  |  |  |  |  |  |  |  |  |  |  |  |
| 2003 | Egypt[47] | Pro | In | All | 97 | 45 | 55 |  | 60-75 | 64.4 |  | 31.0 | 61.0 |  |  | 7.0 |
| * Retrospective, prospective or survey  ** In-patients, outpatients or both. All these studies except Arshi et al[17] are hospital-based.  *** Only deaths included in this study | | | | | | | | | | | | | | | | |
